# The Additions to Radcliffe Infirmary, Oxford

**Published:** 1912-02-17

**Authors:** Edward Warren

**Affiliations:** 20 Bedford Square, London, W.C.


*To the Editor of* The Hospital.

### THE ADDITIONS TO RADCLIFFE INFIRMARY, OXFORD.

*To the Editor of* The Hospital.

Sir,—Will you allow me to point out that in your notice of the additions to the above contained in your last issue you appear to be under a misapprehension in regard to the entrance for patients to the casualty department? They are to be brought up to the door under the covered glass shelter at the south-west angle of the building, whence there is a corridor which will bring them to the casualty room without traversing the out-patients' hall at all, so that you will see that the separate entrance to the casualty room from the outside, which you think it would have been possible to arrange for, has, as a matter of fact, been already provided.

The casualty patients are to be brought direct to the casualty room, and, if requiring a minor operation, are to be placed in the operation room. There are recovery rooms provided for both sexes, so that they can remain for a while before being sent home. This is a special requirement of the Radcliffe Infirmary, many of whose patients come from a distance in the country and have a long way to go to their homes; it is therefore desirable that they should have an opportunity of lying down for some time before being sent out, no matter how trivial the operation may be. I have further to say that the lobbies to the consulting rooms will be amply lit by large ventilating fanlights over the doors. I observe that Dr. Mackintosh's initials are D. J. and not W. J.—Believe me, yours faithfully,

Edward Warren.

20 Bedford Square, London, W.C.

